# TREM-1 as a Potential Coreceptor in Norovirus Pathogenesis: Insights from Transcriptomic Analysis and Molecular Docking

**DOI:** 10.1021/acsomega.4c10220

**Published:** 2025-01-30

**Authors:** Mike Telemaco Contreras Colmenares, Amanda de Oliveira Matos, Pedro Henrique dos Santos Dantas, José Rodrigues Do Carmo Neto, Bruno Júnior Neves, Luiz Gustavo Araújo Gardinassi, Marcelle Silva-Sales, Helioswilton Sales-Campos

**Affiliations:** † Laboratório de Imunologia de Mucosas e Imunoinformática, Instituto de Patologia Tropical e Saúde Pública, Universidade Federal de Goiás, Goiânia 74605-170, Brazil; ‡ Laboratório de Quimioinformática, Faculdade de Farmácia, Universidade Federal de Goiás, Goiânia, GO 74605-170, Brazil; § Escola de Enfermagem de Ribeirão Preto - Universidade de São Paulo, Ribeirão Preto 14040-902, Brazil

## Abstract

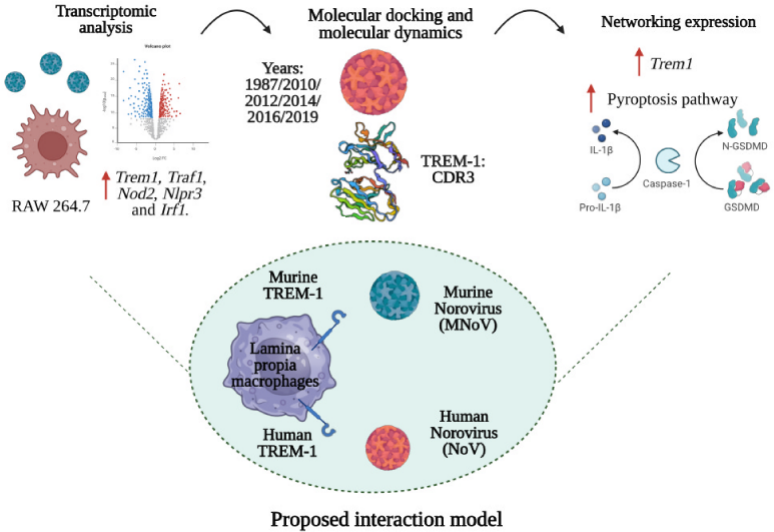

Norovirus (NoV) is a major cause of acute diarrheal disease in humans. However, due to complications in cultivating this virus, bioinformatics aids in elucidating the virus–host relationship. One of the molecules that has been associated with the burden of viral diseases is TREM-1, mainly due to its role in amplifying the inflammatory response. Thus, we hypothesized that TREM-1 may be involved in NoV infection. Analysis of public transcriptomic data sets showed an increased expression of *Trem1* and *Trem3* during murine NoV (MNoV) infection. Then, molecular docking was performed between murine TREM-1 and the P domain of the MNoV VP1 protein. The viral antigenic segment C′–D′ was recognized by the murine TREM-1 CDR1 region. Subsequently, based on phylogenetic criteria, NoV VP1 proteins from the GII.4 genotype sequenced in different years (1987, 2010, 2012, 2014, 2016, and 2019) were modeled. Using docking and molecular dynamics simulations, a stable interaction was observed between the human TREM-1 Ig-like domain and the conserved S and P segments of the NoV VP1 protein. Notably, this interaction was conserved over the years and was mainly dictated by the TREM-1 CDR3 region. Also, coexpression between *Trem1* with genes involved in apoptosis and pyroptosis pathways was surveyed during viral infection by MNoV. It was found that *Trem1* is primarily expressed with genes from the pyroptosis pathway. These simulations strongly suggest the involvement of TREM-1 in NoV pathogenesis and its potential contribution as a coreceptor.

## 1 Introduction

Acute diarrheal disease (ADD) is still a major public health concern worldwide.^1^ Among different causative agents, norovirus (NoV) is one of the major causes of infectious ADD.^2^ The main NoV outbreaks have been reported in low-income regions with inadequate sanitation systems and/or associated with the consumption of poorly sanitized water and food.^3,4^ Even in developed countries like the United States, the NoV can be responsible for up to 60% of ADD cases. Regardless of a country’s economic status, at least 50,000 children die each year due to NoV infection. Despite the high morbidity and mortality rates, there is currently no vaccine available to control this infection.^5^

NoV is an RNA icosahedral virus composed of 180 VP1 proteins, which are encoded in the viral genome’s ORF2. The VP1 protein consists of approximately 530–555 amino acids, with a molecular weight of around 58 kDa.^6^ This protein has two subunits: shell (S) and protruding (P) domains. The P domain is further divided into two subunits, P1 and P2, which provide intermolecular stability among the dimeric subunits of different VP1 units in the viral capsid.^7^ However, variations in the amino acid sequences in different strains in the P2 region dictate the interactions between the virus and host cell receptors. In fact, amino acid substitutions in this region are directly involved in the generation of variants associated with epidemics that occur every 2 to 4 years.^8^ Additionally, because the P2 segment is more exposed on the capsid of the viral particle, it is recognized as the main site for antibody identification directed at the P domain, suggesting that this segment is under constant selective pressure by the immune system.^9^

In NoV strains that infect humans, the VP1 protein binds to its only receptor identified so far, the human histo-blood group antigen (HBGA), which facilitates the interaction between the virus and host cells. Aside from its role in cell adsorption, protein and nonprotein receptors involved in viral infections can also contribute to subsequent responses, including the development of inflammation and cell death.^10^ Specifically, during NoV infection, this interaction has been described as activating proinflammatory pathways, such as pyroptosis and apoptosis, as part of the immunopathological response. In fact, in the RAW264.7 macrophage cell line, infection with murine norovirus (MNoV) induced NLRP3, pro-caspase 1, ASC, cleaved-caspase1, N-GSDMD, IL-1β, and IL-18, known markers of the pyroptosis pathway. Thus, knowing the markers will make it possible to develop treatments aimed at reducing the harmful effects of the virus, such as ADD.^11,12^

As human NoV cultivation is a challenging task for reference laboratories, this has negatively impacted *in vitro* studies and a broader understanding of its biology and pathophysiology.^13^ To minimize this drawback and despite its own limitations, experimental infection with MNoV has been used.^14−16^ Different MNoV strains (CW3, CR6, and S7) can interact with CD300LF, which was recently identified as a physiological receptor for MNoV.^17^ CD300LF is a member of the immunoglobulin superfamily.^18,19^

Pattern recognition receptors (PRR) are known for their ability to interact and recognize microbial (MAMPs) and/or danger/damage (DAMPs) associated molecular patterns. In this regard, based on its structural and phylogenetic similarities to the members of the CD300 family, one PRR deserves special attention, the triggering receptor expressed on myeloid cells-1 (TREM-1), which also belongs to the immunoglobulin superfamily.^20,21^ However, its role in NoV infection has never been addressed before. TREM-1 is expressed both in immune^22−26^ and nonimmune cells^26−30^ and has been implicated in the amplification of inflammation in noninfectious and infectious diseases, including those caused by viruses such as Marburg and Ebola,^31^ human immunodeficiency virus,^32,33^ hepatitis C virus,^34^ dengue virus,^35^ and enterovirus-A71.^36^ In general, higher TREM-1 activity has been linked to greater disease severity and inflammation in the aforementioned scenarios.^37^

Despite the well-documented role of TREM-1 in different viral infections, its contribution to the NoV infection remains to be elucidated. To overcome the limitations in studying the complex relationship between NoV and its hosts, including humans, the use of algorithms and mathematical models as part of bioinformatics workflows can broadly and rapidly predict protein–protein interactions. These predictions can quickly identify interactions that would be difficult to determine *in vitro*, particularly in viruses of public health importance that undergo genetic modifications over time and/or present several limitations in its *in vitro* cultivation, such as NoV. In this study, we used a combination of transcriptomic analysis, molecular docking, and molecular dynamic (MD) simulations as a promising approach to explore the interaction between NoV and TREM-1.^38^ Specifically, we analyzed the expression of *Trem1* and its related genes during the MNoV infection. Then, we conducted *in silico* simulations between murine TREM-1 and the MNoV VP1 protein. Additionally, we evaluated the *in-silico* interactions between human TREM-1 and the GII.4 NoV VP1 proteins isolated from different outbreaks worldwide. Finally, we assessed the stability of these interactions using MD simulations.

## 2 Results

### 2.1 *Trem1* Is Increased during MNoV Infection

**Figure 1:**
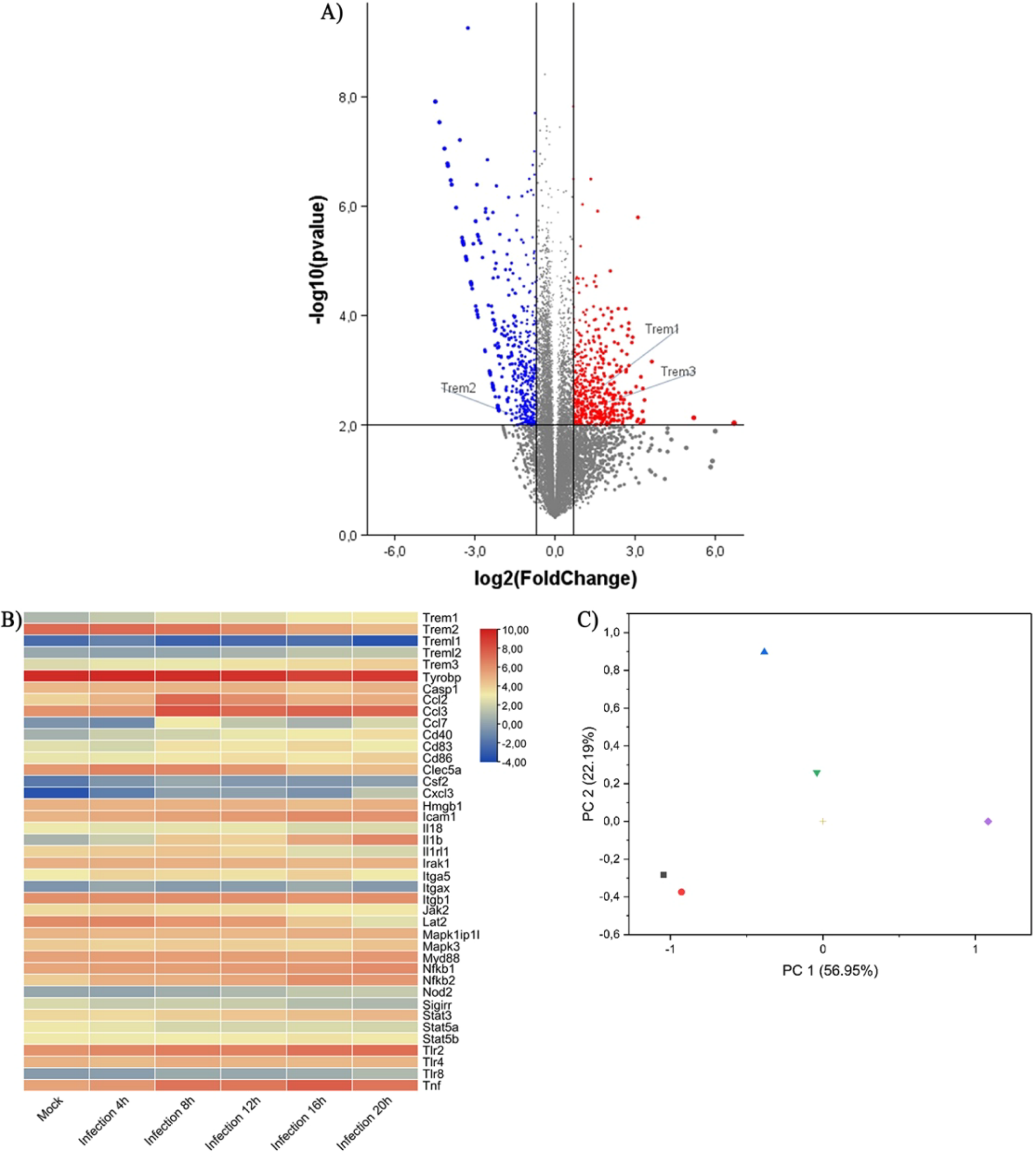
*Trem1* expression increases in the *in vitro* MNoV infection model. (A) The analysis of the entire set of genes from the GSE94821 transcriptomic study showed that *Trem1* and *Trem3* were upregulated during MNoV infection. In contrast, *Trem2* was downregulated. (B) The heat map shows the progressive increase in *Trem1* at different time points. Other inflammation-related genes (*Tnf*, *Icam*, *Il1b*, *Nfkb,* and *Myd88*) were also increased. (C) The principal component analysis (PCA) represents the clustered gene expression of the TREM-1 pathway at different times, these being: the gray square represents grouping the expression of the genes in the mock group. The red circle represents the gene expression at 4 h postinfection; the blue triangle shows the gene expression at 8 h postinfection; the green triangle shows the gene expression at 14 h postinfection; the beige positive sign represents the gene expression at 20 h; and the purple diamond shows the gene expression at 26 h. *Casp1*: caspase 1, *Ccl2*: C–C motif chemokine ligand 2, *Ccl3*: C–C motif chemokine ligand 3, *Cd40*: B cell surface antigen CD40, *Cd83*: cell surface protein HB15, *Cd86*: B-lymphocyte activation antigen B7–2, *Clec5a*: C-type lectin domain containing 5A, *Csf2*: colony stimulating factor 2, *Cxcl3*: C-X-C motif chemokine ligand 3, *Cxcl8*: C-X-C motif chemokine ligand 8, *Hmgb1*: high mobility group box 1, *Icam1*: intercellular adhesion molecule 1, *Il18*: interleukin 18, *Il1b*: interleukin 1 Beta, *Il1rl1*: interleukin 1 receptor like 1, *Il6*: *interleukin*6, *Insig1*: insulin induced gene 1, *Insig2*: insulin induced gene 2, *Irak1*: interleukin 1 receptor associated kinase 1, *Itga5*: integrin subunit alpha 5, *Itgax*: integrin subunit alpha X, *Itgb1*: integrin subunit beta 1, *Jak2*: Janus kinase 2, *Lat2*: linker for activation of T cells family member 2, *Mapk1*: mitogen-activated protein kinase 1, *Mapk3*: mitogen-activated protein kinase 3, *Myd88*: MYD88 innate immune signal transduction adaptor, *Nfkb1*: nuclear factor Kappa B subunit 1, *Nfkb2*: nuclear factor Kappa B subunit 2, *Sigirr*: single Ig and TIR domain containing, *Stat3*: signal transducer and activator of transcription 3, *Stat5a*: signal transducer and activator of transcription 5A, *Stat5b*: signal transducer and activator of transcription 5B, *Tlr2*: toll -like receptor 4, *Tlr4*: toll- like receptor 4, *Tlr8*: toll -like receptor 8, *Tnf*: tumor necrosis factor, *Trem1*: triggering receptor expressed on myeloid cells 1, *Trem2*: triggering receptor expressed on myeloid cells 2, *Treml1*: triggering receptor expressed on myeloid cells like 1, *Treml2*: triggering receptor expressed on myeloid cells like 2, *Tyrobp*: TYRO protein tyrosine kinase-binding protein.

As it has already been observed that *Trem1* expression increases in different viral infections, we sought to investigate transcriptomic public data sets regarding TREM-1 in experimental MNoV infection. Below, we present the genes involved in the expression of the *Trem1* pathway, obtained from transcriptomic study GSE94821. This study was conducted *in vitro* by exposing murine macrophages (RAW 264.7 cells) to the MNoV strain, MNoV-1.^39^ In comparison to *Trem2*, our analysis identified an increased expression of *Trem1* and *Trem3* (Figure 1A). This pattern was also observed for the *Trem1*-related genes in a time-dependent manner (Figure S1). Other inflammation-related genes, such as *Icam1*, *Il1b*, *Tlr2*, and *Tnf*, which are directly associated with TREM-1 activity, were also upregulated (Figure 1B). Also, the genes involved in the TREM-1 pathway were grouped using principal component analysis (PCA) (Figure 1C). During the first 4 h of infection, no differences were observed in the expression of TREM-1 related genes compared to the mock group (simulated viral infection). However, at 8 and 12 h postinfection, the set of genes coding for the TREM-1 pathway showed increased expression compared to the other groups and time points (16 and 20 h) (Figure 1C). Also, the genes encoding proteins for the TREM-1 pathway are upregulated in bone marrow-derived macrophages (BMDM) during MNoV infection (Figure S1).

On the other hand, no differences were detected in the expression of *Trem1* and *Trem*3 in the GSE111642 study^40^ (Figure S1A). Despite this, their data showed a similar pattern to that observed in the GSE94821 study regarding the expression dynamics of *Trem1* during the first 8 h of infection (Figure S1B). In addition, our analysis indicates that *Trem1* expression was associated with greater coexpression with *Traf1*, *Nod2*, *Nlpr3*, *Irf1*, and *Junb* which are genes involved in the antiviral response pathway (Figure S1C). It is important to highlight that we did not find any transcriptomic studies involving NoV infection in human cells, where the expression of *Trem1* was recorded.

### 2.2 The Interaction between Murine TREM-1 and the MNoV VP1 Protein Occurs through the Recognition of a Conserved Domain

As *Trem1* was upregulated during experimental infection in murine macrophages, we first evaluated whether an interaction could exist between TREM-1 and the MNoV VP1 protein. Using the crystal structures of the Ig-like domain of murine TREM-1 and the P domain of MNoV VP1 protein, a molecular docking was performed. Also, we used the interaction between the P domain of the MNoV VP1 protein and the Ig-like domain of murine CD300LF receptor, the putative receptor for MNoV, as a control for our simulations and interaction predictions (Table S1).

**Figure 2:**
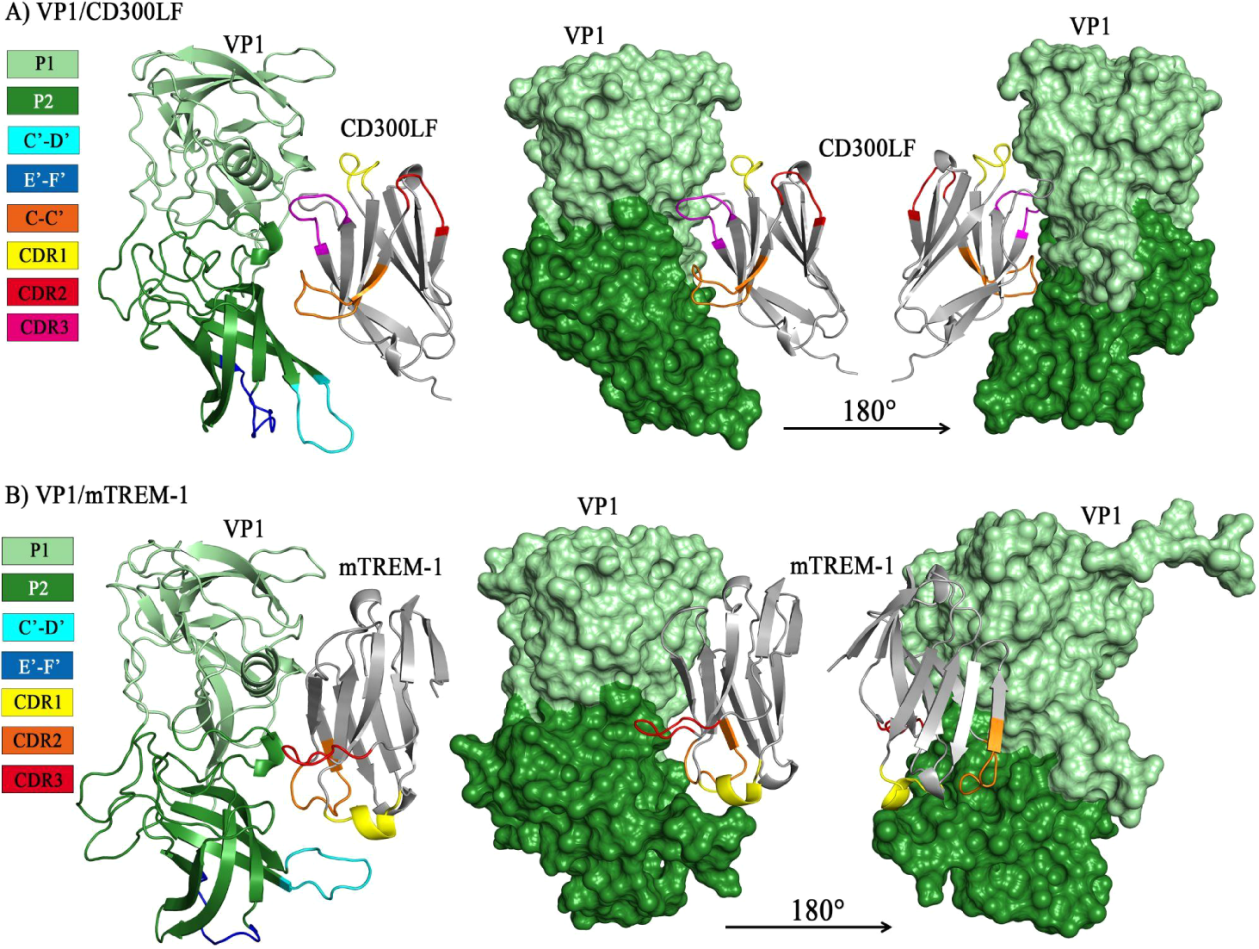
Interaction interface between the structures of murine CD300lf and murine TREM-1 with the MNoV VP1 protein P domain. The protruding (P) domain (amino acids 228–540) of the MNoV VP1 protein (∼540 amino acids) is shown in green (in both A and B), with the P1 domain in light green and P2 in dark green. The antigenic loops of VP1 are also highlighted in cyan (loop C′–D′, amino acids 342–350) and dark blue (loop E′–F′ amino acids 377–388). The IgV-like domains of CD300LF (A) and murine TREM-1 (mTREM-1, B) are depicted in gray. For CD300LF, the C–C′ loop is colored orange, CDR1 region is colored yellow, CDR2 is colored in red, and CDR3 in magenta (A). For mTREM-1, the CDR1 region is highlighted in yellow, CDR2 in orange, and CDR3 in red. (A) The VP1 P2 subdomain is identified by the CD300LF CDR3 region. (B) The C′–D′ antigenic loop of VP1 interacts with CDR1.

The P2 domain of the MNoV VP1 was recognized by the CDR3 and the C–C′ loop of murine CD300LF (Figure 2A), as observed by our molecular docking assay, consistent with those observed in crystal structures and reports in the literature.^41^ Furthermore, we found that the P2 segment of MNoV VP1 was recognized by TREM-1. The CDR1 region made direct contact with the C′–D′ antigenic loop (Figure 2B), which is described as a conserved region of the murine VP1 protein. The possibility that this interaction also occurs *in vivo* was reinforced by the binding free energy (−61.13 kcal/mol). Additionally, the energy values generated by the online servers were within the intervals considered indicative of a reliable result (Table S2).

### 2.3 MD Simulations Suggest That Human TREM-1 Interacts with the NoV VP1 Protein

Based on the recognition of domain P2 of the MNoV VP1 protein by murine TREM-1, we sought to determine whether this interaction could also occur between their human counterparts. We conducted a phylogenetic analysis (Figure S2) to select sequences for protein modeling, molecular docking, and subsequent MD simulations. Given that global outbreaks of ADD caused by NoV occur every 2–4 years, the sequences were chosen based on phylogenetic analysis. The earliest available VP1 NoV sequence in GenBank (ID: ACT76148.1), isolated in 1987 and known as the Bristol strain, was selected. Our phylogenetic analysis also revealed that available sequences between 2000 and 2010 did not show an equitable geographic distribution among common ancestors in the phylogenetic tree. The modeled sequences were used to perform molecular docking simulations: 1987 (GenBank ID: ACT76148.1); 2010 (GB ID: AGC66783.1); 2012 (GB ID: AFV99155.1); 2014 (GB ID: ALQ43926.1); 2016 (GB ID: ANP93428.1); 2019 (GB ID: QEL43936.1). Based on the ranking described in the methodology, the best poses were selected for MD simulations (Table 1). Our ranking system showed that protein–protein MD simulations were robust as the obtained RMSD average values were consistent with the sum of the energy values obtained from each online server. Similarly, this strategy has been successfully used in previous studies.^42,43^ To further confirm the accuracy of our poses under our ranking system, the protein–protein complexes were subjected to a *Z*-score analysis, which determined whether binding occurred between the two complexes. The analysis showed that all complexes exhibited interactions at 4, 6, and 8 Å (Table 1). One possible explanation for the differences in binding estimates at different Angstrom distances is that the *in-silico* predictions do not account for small molecules such as bile acids and cations like calcium and magnesium, which may also influence these interactions. Nevertheless, despite the absence of these elements and molecules, consistent interactions and patterns were observed across all years in which the interaction between TREM-1 and the VP1 protein was evaluated.

**Table 1:** Thermodynamic and Ligation Parameters of the Molecular Docking between Human TREM-1 and the NoV Protein VP1^a^

Isolation year	1987	2010	2012	2014	2016	2019
Pose	Model 6	Model 1	Model 8	Model 6	Model 2	Model 2
Thermodynamic variables						
Lowest energy (kcal/mol)	– 870.4	–838.3	–839.9	–770.9	–851.5	–827.5
Biding affinity (Δ*G*-kcal/mol^–1^)	–14.7	–14.3	–14.8	–14.0	–14.0	–13.4
Binding free energy of complex (kcal/mol)	–101.64	–81.25	–107.17	–113.24	–101.86	–92.76
Total stabilizing energy (kJ/mol)	–444.44	–396.76	–438.31	–481.25	–428.83	–489.20
Predicted binding energy (kcal/mol)	–10.432	–9.849	–10.324	–9.986	–9.338	–9.851
PYDOCK_TOT (−60 to −5)	–50.165	–14.06	–48.502	–46.133	–33.456	–37.577
VDW (−200 to −50)	–139.10	–107.649	–139.078	–124.553	–106.627	–103.682
HBOND (−15 to −1)	–20.07	–82.95	–18.03	–11.8	–15.95	–16.95
FA_ATR (−100 to −20)	–98.158	–38.579	–94.76	–91.849	–83.901	–85.374
ELE (−60 to 0)	–27.035	–19.285	–27.149	–36.442	–24.881	–25.306
DESOLV (−30 to 20)	–9.219	–8.53	–7.446	2.765	2.088	–1.904
Scoring interaction protein complexes (*Z*-score)						
4 Å	2.141 (binder)	1.340 (nonbinder)	1.703 (binder)	1.371 (nonbinder)	2.140 (binder)	1.446 (nonbinder)
6 Å	1.613 (binder)	0.789 (nonbinder)	1.454 (binder)	1.185 (nonbinder)	0.704 (nonbinder)	0.921 (nonbinder)
8 Å	1.861 (binder)	1.032 (binder)	1.614 (binder)	1.414 (binder)	0.754 (nonbinder)	1.170 (binder)
Average of values of molecular dynamics simulations						
RMSD simulation 1 (nm)	0.5427	0.6572	0.5173	0.5857	0.6584	0.5756
RMSD simulation 2 (nm)	0.4885	0.5452	0.6409	0.4908	0.6070	0.5194
RMSD simulation 3 (nm)	0.5085	0.7505	0.7005	0.4497	1.7030	0.4943

a Å: Angstroms. RMSD: root-mean-square deviation. PYDOCK_TOT: total energy. HBOND: hydrogen bond potential. VDW: van der Waals energy. ELE: total electrostatic energy. FA_ATR: attractive van der Waals forces. DESOLV: desolvation energy. nm: nanometers.

**Figure 3:**
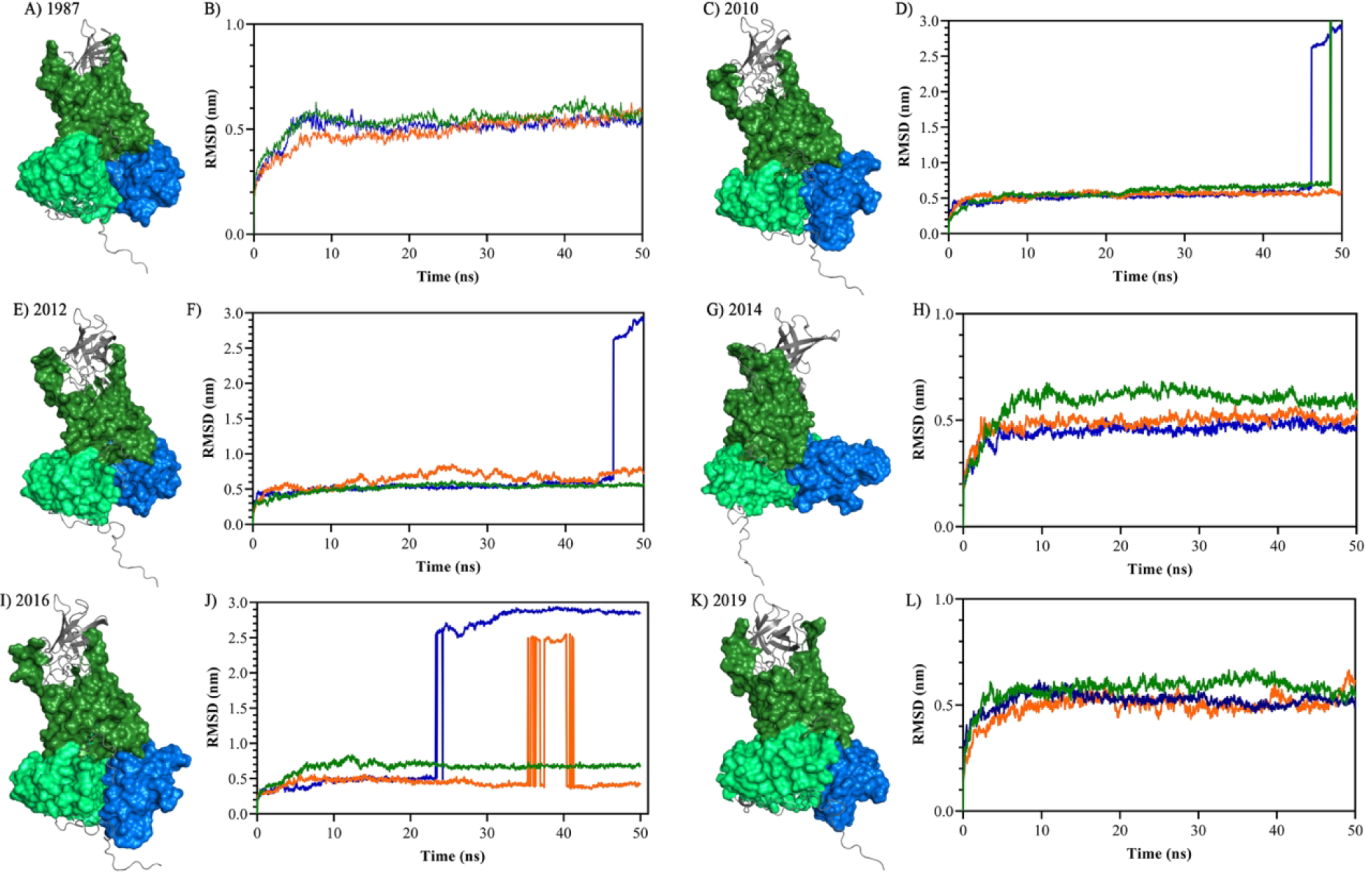
Molecular docking and molecular dynamics simulations show that TREM-1 can interact with the NoVv protein VP1. Initially, molecular docking assays showed that the Ig-like domain of human TREM-1 (dark blue) can interact with the protruding (P)1 (light green) and shell (S) (dark green) domains of the different VP1 modeled proteins (A, C, E, G, I, and K). Simulations showed RMSD values lower than 0.7 nm (B, D, F, H, J, and L), while only one of the replicates from 2016 (J) seemed to be stable. All MD simulations were carried out in triplicates (values of simulations represented by lines in dark blue, dark green, and orange) during 50 ns. RMSD: root-mean-square deviation, ns: nanoseconds, nm: nanometers.

The MD simulations showed low RMSD values within triplicate (Table 1). More specifically, for the years 1987 (Figure 3A), 2014 (Figure 3D), and 2019 (Figure 3F), the interactions remained stable over the 50 ns, thus suggesting a stable interaction between these VP1 proteins and TREM-1. Similarly, the VP1 proteins from 2010 (Figure 3B) and 2012 (Figure 3C) were stable for up to 45 ns of MD simulations. For the VP1 sequence in 2016, only one of the simulations (triplicate) maintained RMSD values below 0.7 for 50 ns. These values are in accordance with the results of the *Z*-score, which allowed for the selection of molecular docking poses.

The binding free energy observed by the HawkDock server reinforces the possibility that the interactions between TREM-1 and the NoV isolates obtained from distinct years can occur in humans. In this context, the binding free energy values varied slightly by year and are presented in descending order according to year and binding free energy values as follows: 2014 (−113.24 kcal/mol), 2012 (−107.17 kcal/mol), 2016 (−101.86 kcal/mol), 2019 (−92.76 kcal/mol), 2010 (−101.64 kcal/mol), and 1987 (−81.25 kcal/mol). These results strongly suggest a link between human TREM-1 and the NoV VP1 protein. The biochemical parameters of the protein–protein interactions showed values within the ranges established by the server (Table 1).

### 2.4 TREM-1 CDRs Recognize Conserved Segments of the VP1 Protein throughout the Years

**Figure 4:**
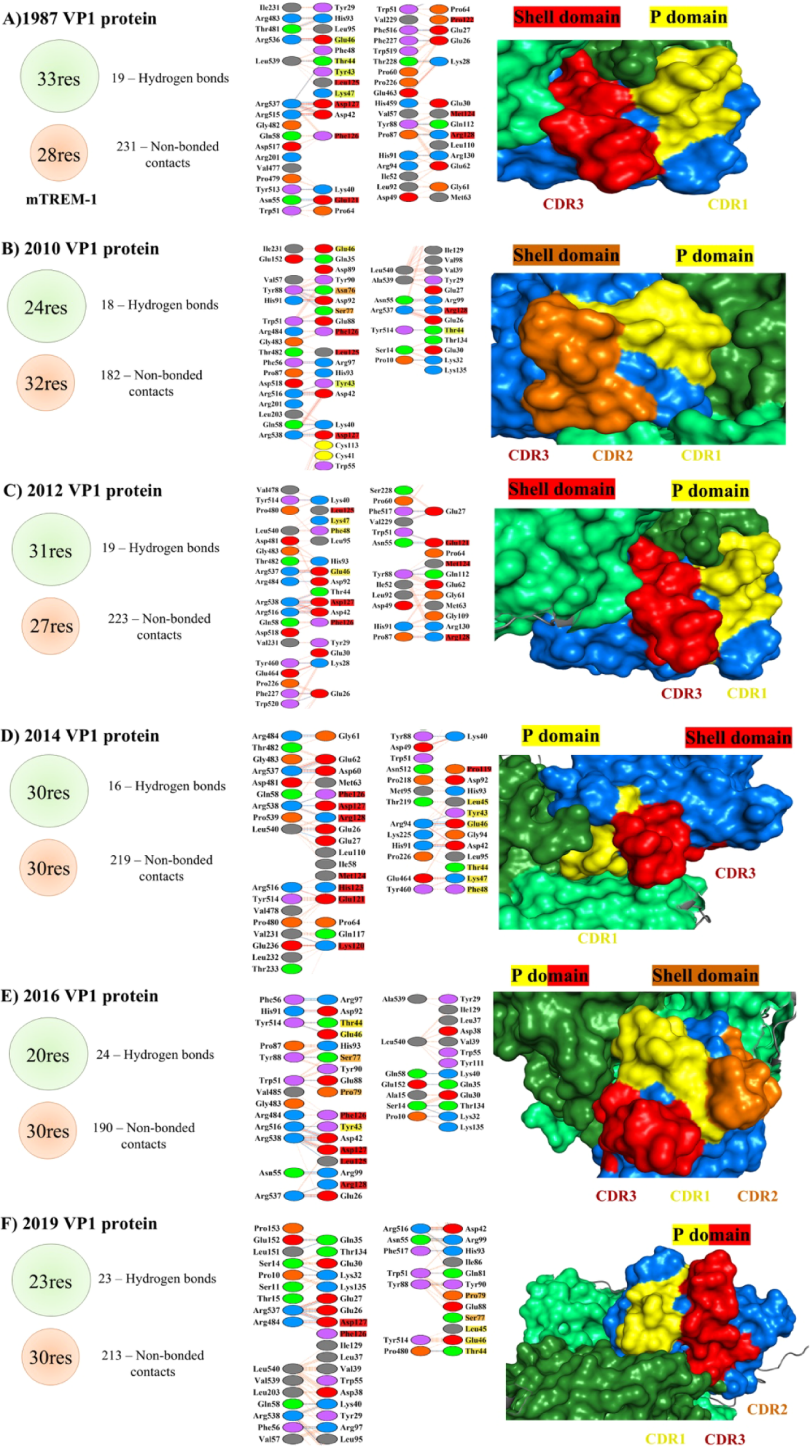
The human TREM-1 CDRs in the interaction with the NoV VP1 protein. The NoV VP1 protein is observed in surface format with the protruding (P) domain in dark green and the shell (S) domain in light green. The Ig domain of human TREM-1 is depicted in dark blue, with the complementary determining regions (CDRs) identified as follows: CDR1 in yellow, CDR2 in orange, and CDR3 in red. Next to the interaction interface, the amino acids present in the CDRs that form hydrogen bonds with the amino acids in the S and P domains of the NoV protein VP1 are highlighted in dark and light green, respectively. Res: residues; Ala: alanine; Arg: arginine; Asn: asparagine; Asp: aspartate; Cys: cysteine; Gln: glutamine; Glu: glutamate; Gly: glycine; His: histidine; Ile: isoleucine; Leu: leucine; Lys: lysine; Met: methionine; Phe: phenylalanine; Pro: proline; Ser: serine; Thr: threonine; Trp: tryptophan; Tyr: tyrosine; Val: valine.

As TREM-1 seemed to interact with the different NoV VP1 proteins, we aimed to determine which TREM-1 CDRs were involved in such interactions. The CDRs involved in the recognition of the conserved S and P1 domains sequenced in different years (1987, 2010, 2012, 2014, 2016, and 2019) varied considerably. Noticeably, the CDR3 region was the most frequently involved throughout the years. The amino acids most frequently detected in these interactions were: CDR1: 42, 43, 44, 46, and 47; CDR2: 76 and 77; and in CDR3: 121, 124, 125, 126, 127, and 128 (Figure 4).

The CDR1 and CDR3 regions were primarily involved in the recognition of NoV VP1 proteins obtained from 1987, 2012, 2014, and 2019 (Figure 4A, C, D,F). The CDR1 region recognized the second portion of the P1 domain, which comprises amino acids 406 to ∼560 (depending on the sequence). In contrast, the CDR3 region recognized the S domain until 2016, and the P1 domain was recognized for the sequences obtained in 2016 (Figure 4E) and 2019 (Figure 4F). On the other hand, the CDR2 region was involved in recognizing the S domain of the VP1 protein only in the sequences obtained from 2010 (Figure 4B) and 2016 (Figure 4E).

**Figure 5:**
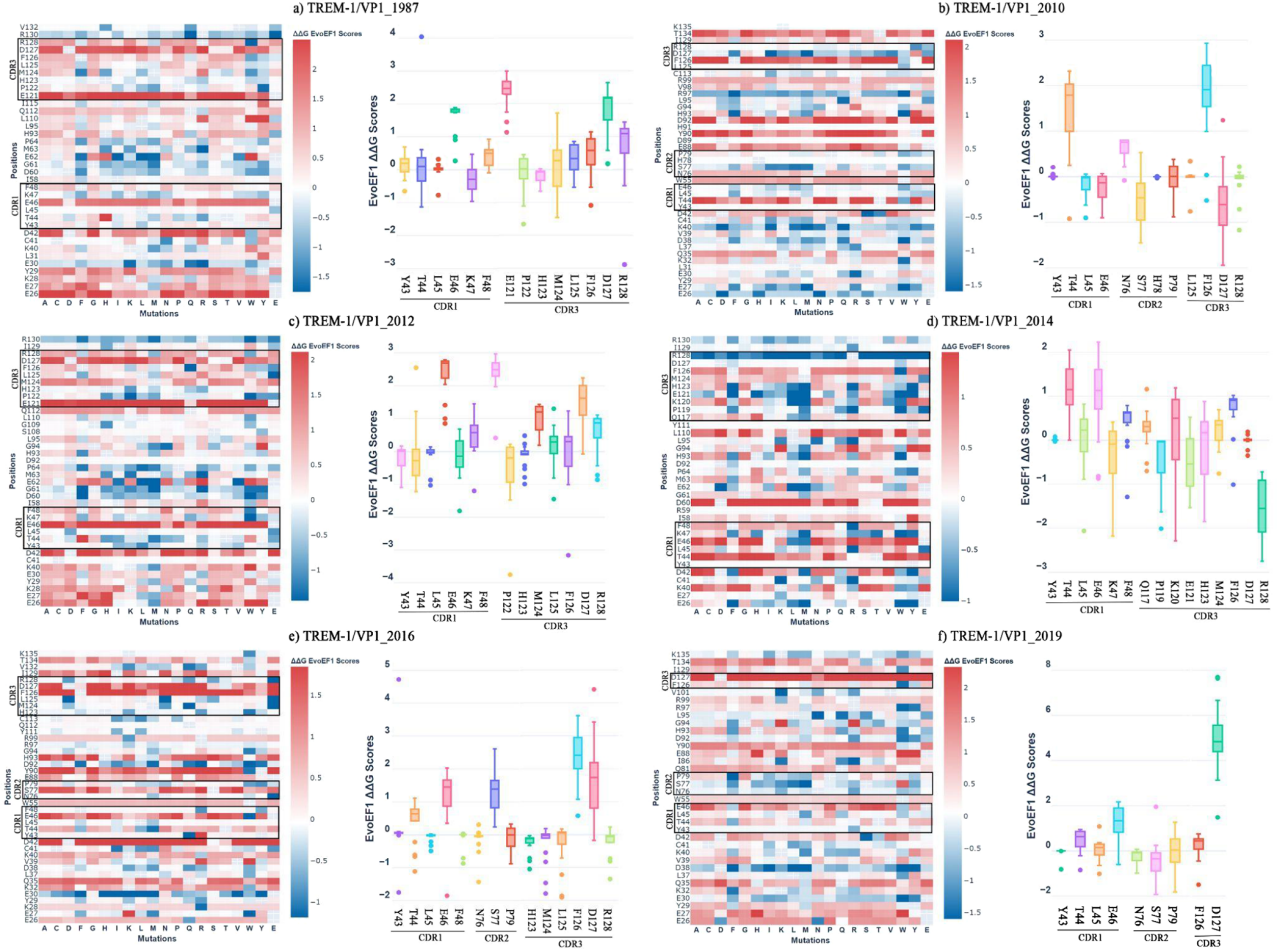
Random mutations in the three CDRs of human TREM-1. Random mutations in the three CDRs of human TREM-1. Heatmaps (left panel) and bar graphs (right panel) show the energetic outcome of different changes in the triggering receptor expressed on myeloid cells-1 (TREM-1) residues, provided by the PROT-ON web server. In the heatmaps, the scale (ΔΔ*G* EvoEF1 scores) indicates that higher intensity values for a residue (red) correspond to lower affinity of the amino acid in the random substitution model due to unfavorable energetic values (positive values). In the bar graphs, the most relevant positions within the CDRs domains of TREM-1 are shown with the variation of the ΔΔ*G* EvoEF1 scores according to the mutation displayed in the bars. The interactions between the receptor and VP1 sequenced in different periods are depicted as follows: (A) TREM-1/VP1_1987, (B) TREM-1/VP1_2010, (C) TREM-1/VP1_2012, (D) TREM-1/VP1_2014, (E) TREM-1/VP1_2016, and (F) TREM-1/VP1_2019.

To confirm the involvement of the human TREM-1 CDRs in the recognition of NoV VP1, we evaluated whether there could be changes in the binding energies in the amino acids that were part of the CDRs by means of a random amino acid substitution method (Figure 5). Regardless of the year of isolation, the random substitution of amino acids in TREM-1 negatively impacted viral recognition, particularly when those mutations occurred in the CDR3 region (Figure 5A–F). Thus, this reinforces the crucial role of this CDR in NoV VP1 recognition. In addition, CDR1 and CDR2 also seemed to contribute to VP1 recognition but to a lesser extent compared to CDR3 (Figure 5). These results suggest a dominant contribution of TREM-1 CDR3 in the recognition of NoV VP1 conserved domains.

### 2.5 *Trem1* Is Coexpressed with Genes Encoding Proteins of the Pyroptosis Pathway

**Figure 6:**
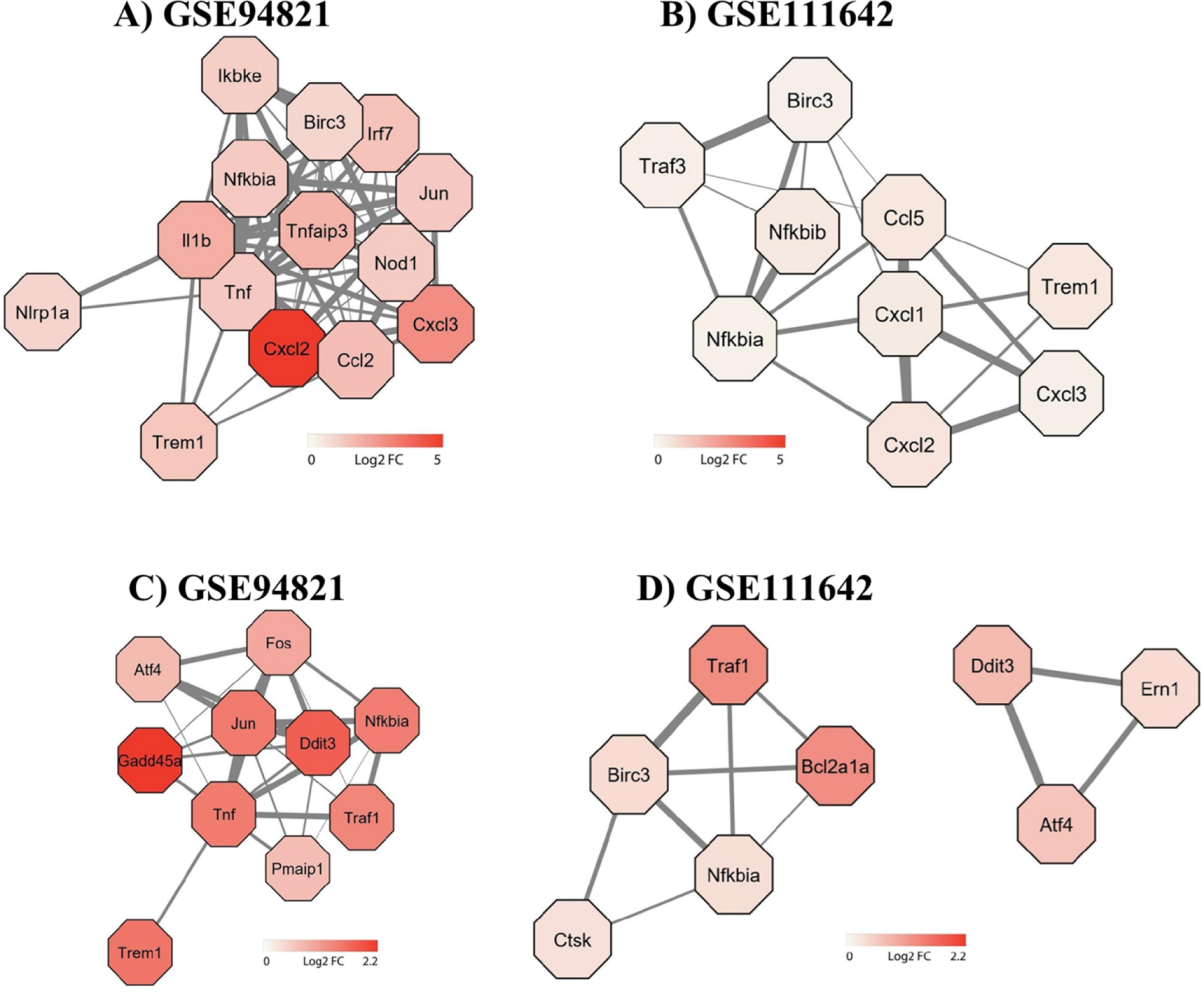
Genes coexpressed with *Trem1* in the pyroptosis and apoptosis pathways in MNoV infection model. Interaction networks were built using data from the GSE94821 and GSE11642 transcriptomics studies. The hexagons identify each gene studied, while the thickness of the gray lines and the intensity of the red color illustrate the degree of association of the coexpression, as determined by the Log2FC value of each gene. The upper line (A and B) shows the coexpression between *Trem1* expression, and the genes associated with the pyroptosis pathway, and the lower line (C and D) shows the coexpression between *Trem1* with the genes associated with the apoptosis pathway. Fos: Fos proto-oncogene, AP-1 transcription factor subunit, Atf4: activating transcription factor 4, Jun: Jun proto-oncogene, AP-1 transcription factor subunit, Traf1: TNF receptor associated factor 1, Ddit3: DNA damage inducible transcript 3, Gadd45a: growth arrest and DNA damage inducible alpha, PAMIP1: phorbol-12-myristate-13-acetate-induced protein 1, Tnf: tumor necrosis factor, Ikbkb: inhibitor of nuclear factor kappa B kinase subunit beta, Birc3: baculoviral iap repeat containing 3, Irf7: interferon regulatory factor 7, Tnfaip3: TNF alpha induced protein 3; Nlpr1: NLR family pyrin domain containing 1, Cxcl3: C–X–C motif chemokine ligand 3, Cxcl2: C–X–C motif chemokine ligand 2, Ccl2: C–C motif chemokine ligand 2, Ccl5: C–C motif chemokine ligand 5, Nod1: nucleotide binding oligomerization domain containing 1, Ern1: endoplasmic reticulum to nucleus signaling 1.

Based on the expression and activity of TREM-1 in the intestine, a major site for NoV infection, and the observation of a dynamic and marked interaction between the receptor and the virus, we sought to understand how this evidence could contribute to local inflammation and disease pathogenesis. For this purpose, the transcriptomic studies GSE94821 and GSE111642, which are based on MNoV infection, were analyzed regarding the coexpression of genes encoding proteins involved in the apoptosis and pyroptosis pathways. The *Trem1* expression was followed by genes encoding chemokines such as *Cxcl2*, *Ccl5*, *Ccl2*, and *Cxcl3*, as well as proinflammatory cytokines such as *Tnf* and *Il1b* (Figure 6A,B), which are more closely associated with pyroptosis (Figure 6A). The relationship between *Trem1* and the genes encoding proteins of the apoptosis pathway was highlighted by the coexpression of *Trem1* and *Tnf* (Figure 6C). Therefore, we can hypothesize that elevated levels of TREM-1 and its related genes during NoV infection are associated with the occurrence of pyroptosis. The activation of this pathway is ultimately implicated in the poorer prognosis, as it increases the production of proinflammatory cytokines, in contrast to apoptosis, where the release of inflammatory mediators by infected cells is low.

## 3 Discussion

Our results suggested the contribution of TREM-1 to NoV pathogenesis alongside its interaction with different VP1 proteins. In general, this receptor interacts with the most conserved regions of MNoV and NoV, indicating a potential role as a viral coreceptor. In humans, this interaction was mainly coordinated by the TREM-1 CDR3 region and was conserved regardless of the year of isolation. Furthermore, the higher expression of *Trem1* observed during *in vitro* infection was associated with genes involved in the pyroptosis pathway rather than apoptosis, strongly suggesting that TREM-1 plays a role in NoV pathogenesis.

Studies aiming at clarifying the NoV–host interaction, particularly those targeting its relationship with the immune system, are crucial for improving our understanding of viral ADD. However, due to several limitations in cultivating NoV, alternative infection models, including murine models and enteroid systems, have been suggested as options to advance knowledge in this area.^44^ Despite these promising alternatives, these methodologies present some constraints, including but not limited to the lack of reproducibility between murine and human infections and the lack of immune cells in studies using enteroids. Thus, additional research methods, such as bioinformatics approaches, are mandatory to elucidate key aspects of the intricate relationship between viruses and their hosts, as well as its implications in disease pathogenesis.^38^

Our approach was initially based on the analysis of public data from transcriptomics studies. Transcriptomics studies in different models of viral diseases^31−37^ have shown that *Trem1* and its related genes directly contribute to the pathophysiology and exacerbation of viral diseases. In our analysis, we observed an increased expression of *Trem1* and *Trem3* in RAW 264.7 cells exposed to an MNoV strain. Our data are in accordance with observations from experimental infections by EV-A71.^36^

Transcriptomic profiling (RNA-seq) of peripheral blood mononuclear cells (PBMCs) demonstrated that genes involved in the TREM-1 activation were upregulated during EV-A71 infection, which was associated with a poor prognosis. Also, it was observed that the increase in *Trem1* expression correlated with higher viral loads and augmented expression of proinflammatory molecules such as *Cxcl3*, *Il1b*, *Il6,* and *Ccl2*. These findings are in accordance with our analysis of *Trem1* expression during the first 8 h of infection with MNoV. Similarly, but using bone marrow-derived macrophages (BMDM), *Trem1* expression peaked during the first 4 h postinfection with MNoV.^45^ These dynamics in TREM-1 gene expression highlight the importance of this receptor in the complex and intricate interface between the host and the NoV.

In fact, the use and interpretation of data from experimental MNoV infections help to elucidate the mechanisms dictating the interactions between NoV and the host. In this context, given the importance of macrophages, monocytes and dendritic cells to intestinal homeostasis, as highlighted in inflammatory diseases,^46^ the analysis of transcriptomic data using macrophages has even greater relevance, particularly for understanding the mechanisms involved in antigen presentation, inflammation, and subsequently, disease outcome.^47^ Under homeostatic conditions, the expression of TREM-1 is low in the intestine and in the lamina propria macrophages.^48^ During homeostasis, these environments tend to be hyporesponsive, a state characterized by elevated local levels of IL-10 and TGF-β, both of which are known as inhibitors of TREM-1.^49^ However, during inflammation, classically activated macrophages in the lamina propria are responsible for increased expression of TREM-1 locally in the intestine, as previously described in experimental models of inflammatory bowel disease.^50,51^ Indeed, intestinal biopsies from pediatric patients have confirmed the presence of the NoV VP1 protein, which is specifically found on the surface of lamina propria macrophages.^52^ In this regard, we strongly believe that the expression and proinflammatory activity of TREM-1 on intestinal macrophages are related to the immunopathogenesis of NoV and may act as a viral coreceptor through the recognition of the antigenic protein VP1. This association stems from the proposal that macrophages in the lamina propria are responsible for primary local viral replication and serve as a reservoir for chronic NoV infection.^53^

In our simulations using the structures of murine TREM-1 and the P segment of the MNoV VP1 protein, we observed an interaction between the P2 segment of the VP1 protein and the CDR1 region of murine TREM-1. A similar behavior was observed in the interaction between MNoV and its most recently described protein receptor, CD300LF, which also belongs to the Ig-like family. It was observed that the P2 segment of MNoV strain CW3 was recognized by the CDR3 loop of murine CD300LF, found in BMDM.^54^ The importance of identifying which CDRs contribute to the interaction is to know how these receptor tertiary structures recognize their ligands and the outcome of this recognition, resulting in immune activation. Despite the similarities between TREM-1 and CD300LF, the differences observed between the studies may, at least in part, be attributed to the different strains used for analysis and the quality of the structures used in each study. In this study, the crystallized P region of the VP1MNV-1 strain was used to simulate the interactions between MNoV and TREM-1. Conversely, in the CD300LF study, the authors used a different strain (CW3), the crystal structure of which is not available in public data sets.

Like what was observed in MNoV infection, human TREM-1 appeared to interact with various modeled VP1 GII.4 proteins of NoV. In all molecular docking and MD simulations, it was consistently observed that the P1 and S segments are recognized. It is important to highlight that these segments do not undergo genetic variability given selective pressure, unlike the P2 segment.^55^ The RMSD values from the MD simulations showed that viral recognition by TREM-1 is stable, with values remaining below 2 nm over 50 ns. Also, although CDRs 1 and 2 participated, the CDR3 region was the most frequently involved in P1 recognition over the years. These findings corroborate previous observations by our group regarding the interaction between TREM-1 and its protein ligands.^56^

Finally, considering that the cytopathic effects produced by NoV infection can activate cell death pathways such as apoptosis and pyroptosis in enterocytes and mucosal gut associated immune cells,^57^ we analyzed the coexpression of *Trem1* and genes involved in the activation of pyroptosis and apoptosis pathways. Our data suggest a stronger association between *Trem1* and the genes encoding proteins of the pyroptosis pathway compared to those involved in apoptosis. This observation was highlighted by the coexpression of *Trem1* with *Il1b*, *Tnf*, *Nod1, Cxcl2*, *Cxcl3*, *Ccl5,* and *Ccl2*. Together, these data suggest a direct contribution of TREM-1 to pyroptosis in MNoV infection. In fact, a previous study demonstrated a direct relationship between MNoV infection, disease outcome, and elevated levels of IL-1β.^58^ In STAT-1 deficient mice, an increased production of IL-1β was observed in the mesenteric lymph nodes and Peyer’s patches in mice experimentally infected by MNoV.^58^ Also, the study detected heightened inflammasome activation, ultimately leading to a greater production of IL-1β and IL-18. The increased production of IL-1β was also associated with increased expression of other inflammatory cytokines, such as IL-18, TNF-α, IL-6, and IL-12p70, probably as an attempt to resolve the infection.^58^ These findings align with the coexpression analysis performed in the present study. This relationship among viral infection, pyroptosis, and the expression of inflammatory cytokines and chemokines has already been described in other viral infections, such as influenza,^59^ the gastrointestinal virus, and rotavirus.^60^

Although this is the first time that TREM-1 has been associated with pyroptosis in viral infections, it has already been described as a potent inducer of pyroptosis in a murine model of chronic obstructive pulmonary disease.^61^ Modulation of TREM-1 using the inhibitory peptide LR17 demonstrated that key pyroptosis-related proteins, such as pro-caspase 1, caspase 1 p10, gasdermin, and gasdermin N, were attenuated following receptor inhibition. In addition, the abrogation of the receptor’s function was associated with a decrease in the production of IL-1β, TNF-α, and IL-18.^61^ Together, these findings align with our data regarding the role of TREM-1 in NoV pathogenesis and infection outcomes.

## 4 Materials and Methods

### 4.1 Transcriptome Data Analysis

The Public Gene Expression Omnibus (GEO) database was surveyed for data sets associated with NoV infection. Data sets that included TREM-1 expression were selected, and data regarding TREM-1 and its related genes were retrieved for multivariate analysis.^62^ For each gene, the FoldChange (FC), the Log2FoldChange (Log2FC), and the *p*-value were calculated using Students’ *t*-test (significant values *p* < 0.05).^63^ The TBtools^64^ software was used with normalized values to create volcano plots and heatmaps. Principal component analysis (PCA) graphs were built using OriginPro 2023b (Origin Lab Corporation, USA, RRID: SCR_014212). Cytoscape was used to construct maps regarding the coexpression of *Trem1* with genes related to apoptosis and pyroptosis (https://cytoscape.org/).^65^ We also considered evaluating the expression of other important members of the TREM family such as *Trem2* and *Trem3*. The inclusion of these two other genes was justified by the fact that TREM-2 is generally described with anti-inflammatory activity,^66^ while TREM-3 activity was described only in mice, with similar activity to that observed in TREM-1.^67^

### 4.2 Modeling of 3D Structures VP1 Protein of NoV

As no crystal structures of human NoV were found in public protein structure databases, including Protein Data Bank (PDB) RCSB (https://www.rcsb.org/) and PDB in Europe (European Bioinformatics Institute, https://www.ebi.ac.uk/pdbe/); and given the high diversity of NoV among its genogroups and genotypes, we searched for known and reviewed genetic sequences coding for the VP1 NoV protein from genotype II (GII.4) at the NCBI (https://www.ncbi.nlm.nih.gov/nuccore). For the final selection, the following criteria were used for the sequences obtained: 1) complete sequences of ORF2-VP1-NoV-GII.4 genes (∼1600 bp); 2) gene sequences with detailed information (region, year, and strain); 3) a clonal representative that is part of a study group; and 4) complete sequence. Out of 1,553 available NoV GII.4 VP1 sequences, only 43 met these criteria. After preliminary analysis, the sequences were divided into three groups: the first group included samples obtained prior to the year 2000; the second group contained samples sequenced between 2000 and 2010; and the third group included sequences obtained after 2010. The sequences were then aligned using the ClustalW method, which performs multiple alignments with 2,000 bootstraps to calculate sequence similarity, via the sequence alignment editor BioEdit. Then selected sequences were used to model protein structures in their tertiary conformation.^68^

To obtain representative sequences from each phylogenetically distinguished group, we performed a phylogenetic analysis using the Molecular Evolutionary Genetics Analysis program—MEGA 11.10.1 (https://www.megasoftware.net/).^69^ Initially, the program was set to identify the best study model for the protein sequence using maximum likelihood (ML), which is a default operation performed by the program. The ML statistical method^70^ was used to build the phylogenetic tree, with 500 bootstrap estimates for the phylogenetic test. Subsequently, the tertiary structures of the VP1 proteins from each year were predicted using the selected amino acid sequences via the AlphaFold2 server through ColabFold v1.5.2,^71^ using amino acid sequences derived from the UniProt database (https://www.uniprot.org).^72^ All three-dimensional structures were analyzed using the MolProbity server to assess the quality of the modeled structures.^73^

### 4.3 Molecular Docking

Rigid molecular docking was performed using the ClusPro online server (https://cluspro.bu.edu/signup.php).^74^ To analyze the interactions between MNoV VP1 and murine TREM-1, PDB files containing the crystal structures were **6C6Q** for accessing the P domain of MNoV-1 CW3 VP1, considering the P1 domain comprised residues R229 to L276 and P415 to S533, while the P2 domain included residues T277 to V413.^54^**1U9K** was used to refer to the three-dimensional structure of murine TREM-1, identifying the complementarity determining regions (CDR) as follows: CDR1 (N44–N50); CDR2 (Q71–Q79); and CDR3 (Y117–V124).^75^ To access the structure of CD300LF, we use the PBD file **6C6Q,** which contains a chain of the CMRF35-like molecule 1,^54^ considering the CDRs within this structure as follows: CDR1 (T25–Y31), CDR2 (D51–L56), and CDR3 (T93-M100).^76^

Similarly, the **1SMO** (resolution: 1.47 Å)^77,78^ structure of human TREM-1 was used to assess the interactions between the receptor and the following modeled structures of VP1 NoV GII.4, which infect humans: Hu/GII.4/CHDC4108/1987/US, GenBank: ACT76148.1; Hu/GII.4/GZ2010-L26/Guangzhou/CHN/2010, GenBank: AGC66783.1; Hu/GII.4/Hong Kong/CUHK3655/2012/CHN, GenBank: AFV99155.1 2012; GII.4 strain GII.4/142696/Shanghai/2014/CHN, GenBank: ALQ43926.1 2014; GII.4/2016, GenBank: ANP93428.1; and GII.4/2019, GenBank: QEL43936.1. The complementarity-determining region (CDR) residues of human TREM-1 were located at the following positions: CDR1: 43–52, CDR2: 70–79, and CDR3: 116–128.

The 10 best-ranked poses were then reranked according to an in-house protocol. Briefly, the 10 poses were analyzed based on their energetic parameters and the thermodynamic values using the Prodigy web server to calculate binding affinity (Δ*G*-kcal/mol^–1^) (https://wenmr.science.uu.nl/prodigy/);^78^ area-affinity Web server to obtain the predicted binding affinity (kcal/mol) (https://affinity.cuhk.edu.cn);^79^ the PPCheck Web server to calculate the total stabilizing energy (kJ/mol) (http://caps.ncbs.res.in/ppcheck/);^80^ and the HawkDock Web server to estimate the binding free energy of the complex (kcal/mol) (http://cadd.zju.edu.cn/hawkdock/).^81^ Values ranging from 1 to 10 were assigned based on the descending order of energy values, with the highest negative energies receiving lower numbers. This assignment was initially determined by the Cluspro ranking position and subsequently by the lowest energy of each prediction (measured in kcal/mol). Finally, the sum for the six different values (Cluspro position rank; Cluspro lowest energy; prodigy binding affinity; area-affinity predicted binding affinity; PPCheck total stabilizing energy; HawkDock binding free energy) was calculated. The pose with the highest cumulative score was then selected for the MD simulation.

### 4.4 Molecular Dynamics (MD) and Computational Characterization of Protein–Protein Interactions

For MD simulations, the WebGro server (https://simlab.uams.edu/index.php) was employed, which is based on GROMACS software.^82^ The MD was performed with the GROMOS 43a1 force field, in a triclinic box with the SPC water model and neutralized with 0.15 M NaCl. Energy minimization utilized a steepest descent integrator with 10,000 steps. The equilibration of the number of particles (*N*), system volume (*V*), and temperature (*T*) [*NVT*] and number of particles (*N*), system pressure (*P*), and temperature (*T*) [*NPT*] was conducted at 310 K. Pressure was set at 1 bar with 50 ns of simulation, generating approximately 1,000 frames per simulation. Each complex underwent triplicate simulations. Graphs were generated using GraphPad Prism version 8.0, utilizing output data from WebGro for visualization and analysis.

The computational characterization of protein–protein interactions was performed using the CCharPPI Web server (http://caps.ncbs.res.in/ppcheck/).^83^ Then, the following descriptors were analyzed: PYDOCK_TOT (total energy), HBOND (hydrogen bond potential), VDW (van der Waals energy), ELE (total electrostatic energy), FA_ATR (attractive van der Waals forces), and DESOLV (desolvation energy).^84−87^ The protein–protein interfaces were generated using the PDBsum online server (https://www.ebi.ac.uk/thornton-srv/databases/pdbsum/Generate.html) to verify complex hydrogen bonds and nonbonded contacts.^88^ Likewise, the results from the aforementioned server were corroborated with those obtained from PPCheck.^83^ Also, to confirm whether these interactions occurred within the entire complex, the PIZSA server (http://cospi.iiserpune.ac.in/pizsa/) was used to determine its potential as either a binding (stable) or nonbinding (unstable) complex.^89^ Finally, to assess whether the amino acid residues that formed part of the CDRs involved in identifying the viral protein VP1 were significant in this interaction, they were subjected to random mutation analysis using the PROT-ON web server (http://proton.tools.ibg.edu.tr:8001/new-run).^90^

## 5 Conclusions

Despite the limitations imposed by the cultivation of NoV in traditional cell culture systems, the use of bioinformatics tools enabled us to explore the contribution of TREM-1 to the recognition of NoV antigens, particularly the VP1 protein. Additionally, our results strongly suggest a direct role for TREM-1 in NoV pathogenesis, as well as its potential function as a coreceptor during this viral infection.

## Supporting Information Available

Table S1: protein characteristics of murine TREM-1 and MNoV VP1; Table S2: molecular docking parameters between murine TREM-1, CD300LF, and MNoV VP1; Figure S1: transcriptomic data of *Trem1* expression in bone marrow derived macrophages from MNoV infected mice; Figure S2: phylogenetic tree of the genetic sequences of human NoV used in this study (PDF)
