# Supplementary material for: TREM-1 as a Potential Coreceptor in Norovirus Pathogenesis: Insights from Transcriptomic Analysis and Molecular Docking
Source: ACS Omega. 2025 Jan 30;10(5):4881–95. doi: 10.1021/acsomega.4c10220 (PMC11822722; doi:10.1021/acsomega.4c10220)
Supplement: Supplementary file 1 — ao4c10220_si_001.pdf [file ao4c10220_si_001.pdf]

TREM-1 as a Potential Co-receptor in Norovirus Pathogenesis: Insights from Transcriptomic Analysis and Molecular Docking

Mike Telemaco Contreras Colmenares[1#], Amanda de Oliveira Matos[1#], Pedro Henrique dos Santos Dantas[1], José Rodrigues do Carmo Neto[1], Bruno Júnior Neves[2], Luiz Gustavo Araújo Gardinassi[3], Marcelle Silva-Sales[1#], Helioswilton Sales-Campos[1#*]

[1] Laboratório de Imunologia de Mucosas e Imunoinformática, Instituto de Patologia Tropical e Saúde Pública, Universidade Federal de Goiás, Goiânia, Brazil.

[2] Laboratório de Quimioinformática, Faculdade de Farmácia, Universidade Federal de Goiás, Goiânia, GO, Brasil.

[3] Escola de Enfermagem de Ribeirão Preto - Universidade de São Paulo, Brazil.

Supporting Information

Supplementary Tables

Table S1. Selected proteins of murine origin for molecular docking

Parameters	TREM-1	VP1
**PDB ID**	1U9K	6C6Q
**Reference**	Kelker et al., 2004	Nelson et al., 2018
**Size**	25 – 133	229–531
**Method**	X-ray diffraction	X-ray diffraction
**Resolution**	1.76 Å	2.00 Å
**Protein segment**	Ig *like*	P domain

PDB ID: Protein Data Bank identification. Å: Angstrom.

Table S2. Molecular docking between murine TREM-1 and CD300LF with P domain of MNoV VP1 protein

Parameters	Murine CD300LF/VP1 MNoV	Murine TREM-1/VP1 MNoV
**Cluster size** (N)	107	74
**Average energy**	-603.7	-660.4
**More negative energy**	-672.9	-741.3
**Binding free energy** (kcal/mol)	-53.68	-61.13
**PYDOCK_TOT** (-60 a -5)	-17.177	-32.42
**VDW** (-200 a -50)	-96.623	-117.174
**HBOND** (-15 a -1)	-11.7	-6.98
**FA_ATR** (-100 a -20)	-60.321	-63.316
**ELE** (-60 a 0)	-14.506	-12.407
**DESOLV** (-30 a 20)	6.99	-8.298

PYDOCK_TOT: total energy. HBOND: hydrogen bond potential. VDW: Van der Waals energy. ELE: total electrostatic energy. FA_ATR: attractive van der Waals forces) and DESOLV: desolvation energy. N: number.

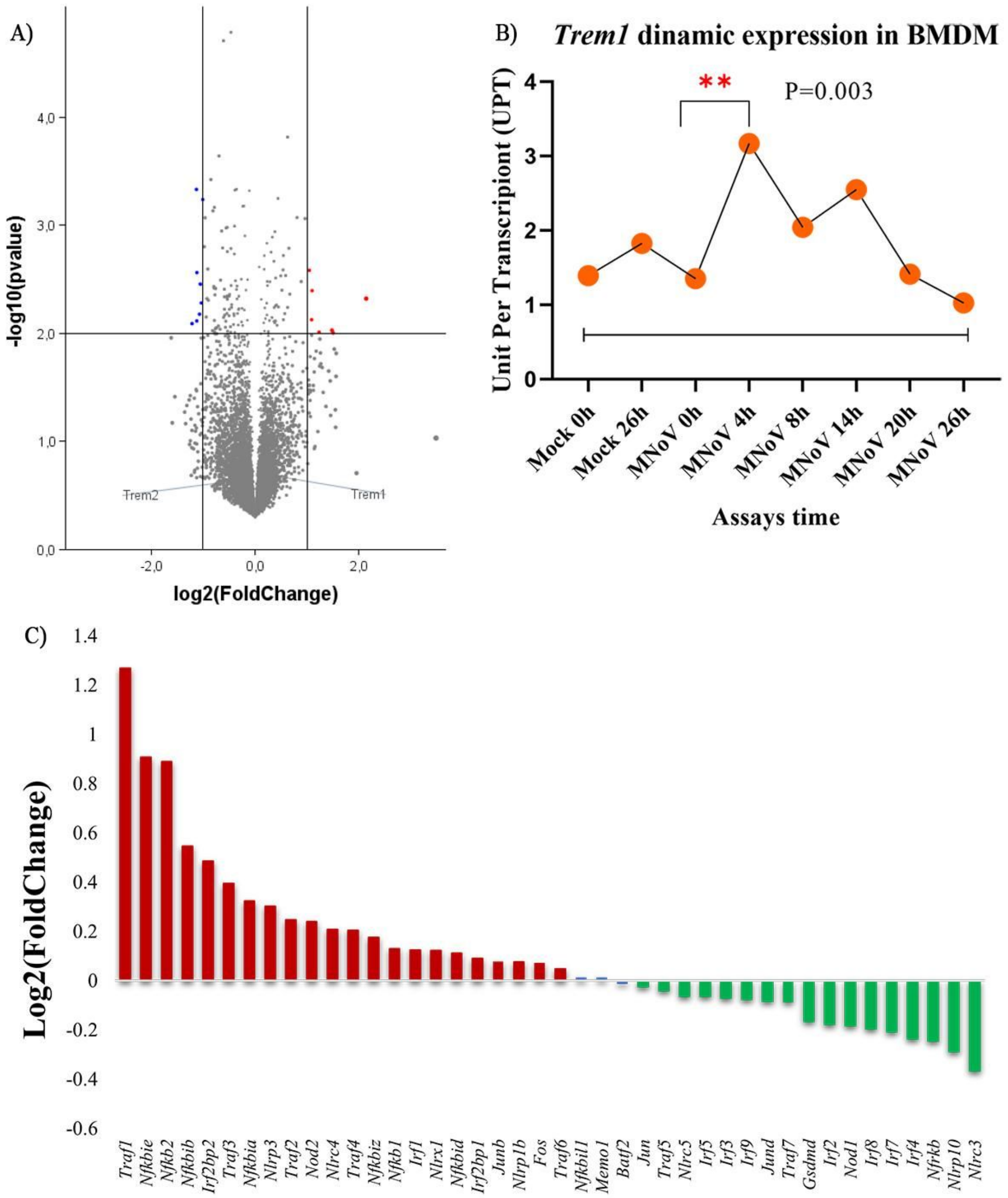

Figure S1. Transcriptional analysis of two genes associated with the expression of *Trem1* and other genetic markers in MNoV infection in bone marrow-derived macrophages (BMDM) of BALB/c mice. A. No significant increase or decrease was observed in the expression of *Trem1* and *Trem2*, when compared with the set of genes obtained in the transcriptomic study of bone marrow-derived macrophages (BMDM). B. The analysis of *Trem1* expression dynamics showed that its expression is higher between the first 4 hours postinfection, with a significant value ($p<0.050$). C. Antiviral response genes are shown. Those with increased expression are identified in red, and those with decreased expression are identified in green. Genes identified in red are associated with increased *Trem1* expression. *Batf2*: basic leucine zipper ATF transcription factor. *Fos*: Fos proto-oncogene of the

AP-1 Transcription Factor Subunit. *Gsdmd*: gasdermin D. *Irf1*: interferon regulatory factor 1. *Irf2*: Interferon regulatory factor 2. *Irf2bp1*: Interferon regulatory factor binding protein. *Irf2bp2*: interferon regulatory factor 2 binding protein. *Irf3*: interferon regulatory factor. *Irf4*: interferon regulatory factor 4. Irf5: interferon regulatory factor 5. *Irf7*: interferon regulatory factor 7. *Irf8*: interferon regulatory factor 8. *Irf9*: interferon regulatory factor 9. *Jun*: Jun proto-oncogene, from the AP-1 transcription. *JunB*: subunit of the AP-1 transcription factor. *Jund*: JunD proto-oncogene, subunit of the AP-1 transcription factor. *Memo1*: mediator of cell motility 1. *Nfkb1*: B lymphocyte nuclear factor κ subunit 1. *Nfkb2*: B lymphocyte nuclear factor κ subunit 2. *Nfkbia*: NF-κB inhibitor α. *Nfkbib*: NF-κB β inhibitor. *Nfkbid*: NF-κB inhibitor δ. *Nfkbie*: NF-κB inhibitor ε. *Nfkbil1*: NF-κB-like inhibitor 1. *Nfkbiz*: NF-κB ζ inhibitor. *Nfrkb*: protein that binds to nuclear factor κ of B lymphocytes. *Nlrc3*: protein 3 of the NLR family that has a CARD domain. *Nlrc4*: NLR family CARD domain containing 4. *Nlrc5*: domain 5 containing an NLR family CARD domain. *Nlrp10*: protein 10 that has a PYRIN domain that belongs to the NLR family. *Nlrp1b*: 1b protein that has a PYRIN domain that belongs to the NLR family. *Nlrp3*: protein 3 that has a PYRIN domain that belongs to the NLR family. *Nlrx1*: X1 member of the NLR family. *Traf1*: factor associated with the TNF1 receptor. *Traf2*: factor associated with the TNF receptor 2. *Traf3*: factor associated with the TNF3 receptor. *Traf4*: factor associated with the TNF4 receptor. *Traf5*: TNF receptor-associated factor 5. *Traf6*: TNF receptor-associated factor 6. *Traf7*: TNF receptor-associated factor7.

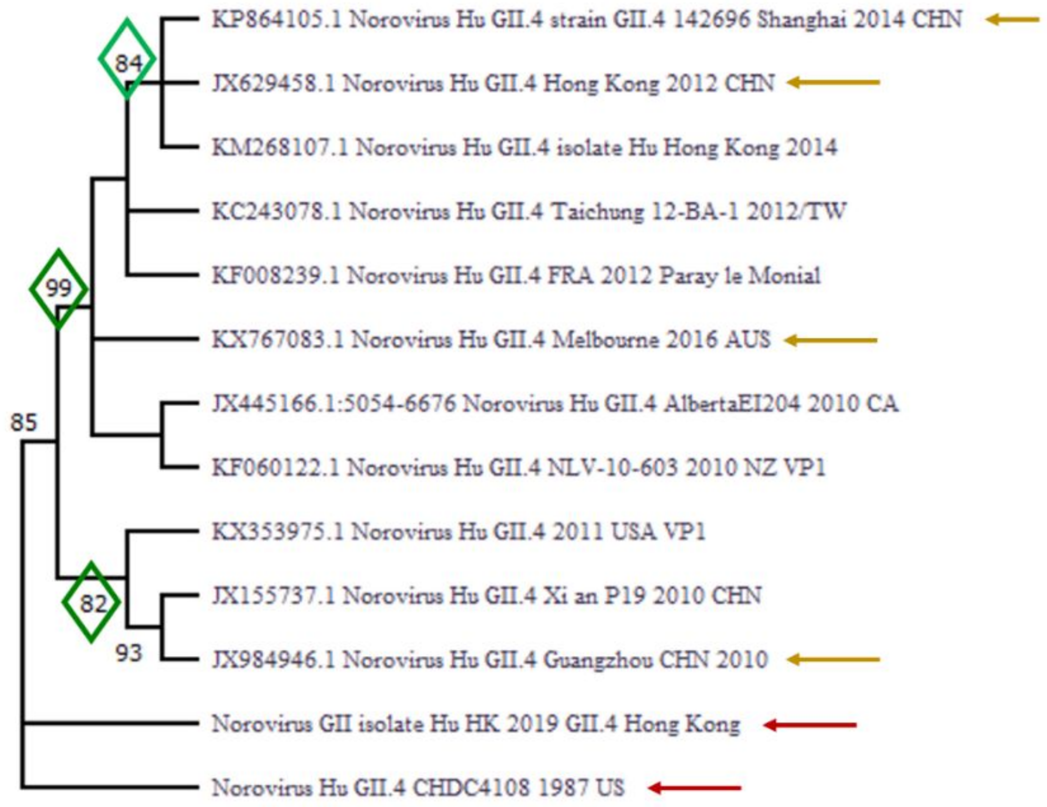

Figure S2. Phylogenetic tree of the genetic sequences that encode the VP1 protein of NoV GII.4. Algorithms were used to create a matrix of pairwise distances estimated using the JTT model. A discrete range distribution was used to model the differences in evolutionary taxa between locations (5 categories (+G, parameter = 0.1625)). This analysis involved 13 amino acid sequences. There are a total of 540 positions in the final dice set.
